# Computed Tomography Features of Incidentally Detected Diffuse Thyroid Disease

**DOI:** 10.1155/2014/921934

**Published:** 2014-12-08

**Authors:** Myung Ho Rho, Dong Wook Kim

**Affiliations:** ^1^Department of Radiology, Kangbuk Samsung Hospital, Sungkyunkwan University School of Medicine, Seoul 110-746, Republic of Korea; ^2^Department of Radiology, Busan Paik Hospital, Inje University College of Medicine, Busan 614-734, Republic of Korea

## Abstract

*Objective*. This study aimed to evaluate the CT features of incidentally detected DTD in the patients who underwent thyroidectomy and to assess the diagnostic accuracy of CT diagnosis. *Methods*. We enrolled 209 consecutive patients who received preoperative neck CT and subsequent thyroid surgery. Neck CT in each case was retrospectively investigated by a single radiologist. We evaluated the diagnostic accuracy of individual CT features and the cut-off CT criteria for detecting DTD by comparing the CT features with histopathological results. *Results*. Histopathological examination of the 209 cases revealed normal thyroid (*n* = 157), Hashimoto thyroiditis (*n* = 17), non-Hashimoto lymphocytic thyroiditis (*n* = 34), and diffuse hyperplasia (*n* = 1). The CT features suggestive of DTD included low attenuation, inhomogeneous attenuation, increased glandular size, lobulated margin, and inhomogeneous enhancement. ROC curve analysis revealed that CT diagnosis of DTD based on the CT classification of “3 or more” abnormal CT features was superior. When the “3 or more” CT classification was selected, the sensitivity, specificity, positive and negative predictive values, and accuracy of CT diagnosis for DTD were 55.8%, 95.5%, 80.6%, 86.7%, and 85.6%, respectively. *Conclusion*. Neck CT may be helpful for the detection of incidental DTD.

## 1. Introduction

Thyroid disease is classified into nodular and diffuse types, and diffuse thyroid disease (DTD) includes autoimmune and nonautoimmune thyroid disease [1]. Autoimmune thyroid disease includes conditions such as Hashimoto thyroiditis, chronic lymphocytic thyroiditis, Graves' disease, and silent/painless/postpartum thyroiditis, and nonautoimmune thyroid disease includes conditions such as acute thyroiditis, subacute thyroiditis, and diffuse hyperplasia [1–3]. DTD is a major cause of thyroid dysfunction; Graves' disease and silent thyroiditis are usually associated with thyroid hyperfunction, while Hashimoto thyroiditis is typically associated with hypofunction [1]. Clinicoserologic detection of thyroid dysfunction is well established, but clinicoserologic detection of asymptomatic or subclinical DTD is not easy [4–10]. Recently, a study using real-time thyroid ultrasound (US) demonstrated that thyroid US is a useful tool for detecting DTD [4], and these imaging-based diagnoses may be helpful in the management of asymptomatic or subclinical thyroid dysfunction.

Currently, computed tomography (CT) is used for TNM staging in thyroid cancer patients, while thyroid US is accepted as the first choice in the diagnosis of thyroid lesions. Despite the radiation hazard, neck CT is a popular imaging tool for evaluating various neck lesions because it has many advantages, such as a wide field of view, objectivity, and detailed display of bone or air-containing organs [5, 6]. To the best of our knowledge, no previous study regarding CT features in the detection of incidental DTD has been published. Therefore, the purpose of this study was to assess CT features suggestive of DTD and diagnostic accuracy of CT diagnosis in patients who underwent thyroidectomy.

## 2. Materials and Methods

### 2.1. Study Population

This study was approved by the Institutional Review Board before subject selection began (KBC13098) and informed consent was waived for this retrospective study. From July 2012 to December 2012, CT scans of the neck were performed preoperatively in 218 patients (177 women and 41 men; average age 47.0 ± 10.3 years; age range 22–75 years) scheduled for surgical treatment of thyroid malignancy or other thyroid lesions. Exclusion factors for this study included neck CT showing previous thyroidectomy or other neck operations and poor image quality. Ultimately, 9 patients (7 women and 2 men; average age 44.5 ± 10.4 years; age range 31–61 years) were excluded from the study.

### 2.2. Neck CT

Neck CT scans were conducted using a multidetector CT scanner (iCT SP 128; Philips Medical Systems, Cleveland, OH) with intravenous injection of contrast medium (120 mL iopromide (Ultravist 300); Bayer HealthCare Pharm., Wayne, Germany; 3 mL/s and 2 mL/Kg). Nonenhanced axial, contrast-enhanced axial, and contrast-enhanced coronal reformatted CT images were acquired in all cases (slice thickness, 3 mm; reconstruction increment, 3 mm; 250 mA, 120 KVp; 140–300 mA). The mean time interval between preoperative neck CT and thyroid surgery was 6.5 days (range 2–15 days).

### 2.3. Image Analysis

**Figure 1:**
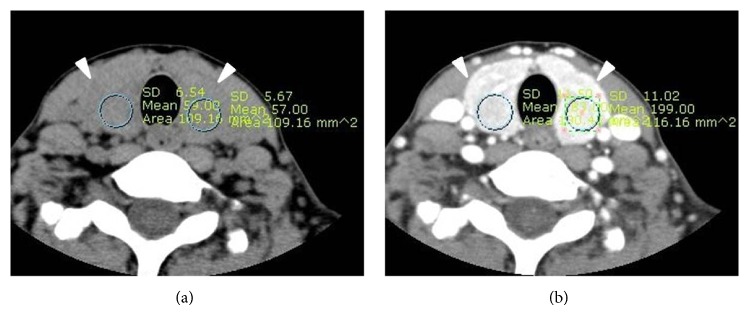
A measurement method of parenchymal Hounsfield unit (HU) by using regions of interest (ROI) (arrows) in nonenhanced (a) and contrast-enhanced (b) CT images.

A single radiologist with 17 years' experience in head and neck CT interpretation performed image analysis with a picture archiving and communication system (PACS). The CT features of the thyroid gland were retrospectively investigated on the basis of the degree and pattern of parenchymal attenuation, glandular size and margin, and degree and pattern of parenchymal enhancement. For the degrees of parenchymal attenuation and enhancement, Hounsfield unit (HU) values were measured in both thyroid lobes, by using regions of interest (ROIs) on nonenhanced and contrast-enhanced CT images, respectively, and then averaged (Figure 1). The difference of HU values between nonenhanced and contrast-enhanced CT was determined as the degree of parenchymal enhancement. Both glandular attenuation and enhancement were categorized into homogeneous, inhomogeneous, and heterogeneous patterns. The anteroposterior diameters of both lobes of the main thyroid were measured in contrast-enhanced CT images, averaged, and classified into 3 categories: 1-2 cm (normal), <1 cm, and >2 cm. The margin of the thyroid was classified as smooth or lobulated.

### 2.4. Histopathology of Thyroid Parenchyma

Histopathological classification was carried out by a single pathologist according to the following criteria: (1) Hashimoto thyroiditis showed progressive loss of thyroid follicular cells with replacement by lymphocytes and formation of germinal centers associated with fibrosis; (2) non-Hashimoto lymphocytic thyroiditis displayed diffuse infiltration of the thyroid gland with lymphocytes and other inflammation-related cells, with none of the typical histopathologic findings of Hashimoto thyroiditis, such as oxyphilic metaplasia, follicular atrophy, and follicular disruption; (3) diffuse hyperplasia showed diffuse hypertrophy and hyperplasia of follicular cells with retention of lobular architecture and no definite nodule formation; (4) normal thyroid showed that there was no visual evidence of coexisting diffuse thyroid disease.

### 2.5. Statistical Analysis

The chi-square test was used to compare the individual items (degree and pattern of attenuation, glandular size and margin, and degree and pattern of parenchymal enhancement) between normal thyroid and DTD. The sensitivity, specificity, positive and negative predictive values, and accuracy of CT for diagnosis of DTD were calculated. Logistic regression analysis was performed to determine cut-off HU values for parenchymal attenuation and enhancement. The highest diagnostic accuracy of CT diagnosis according to the cut-off HU value and CT classification system was determined by receiver-operating characteristic (ROC) analysis. To evaluate the efficacy of the cut-off HU values of the thyroid parenchyma and the CT classification system for differentiation between normal thyroid and DTD, the areas under the ROC curve (*A*_*z*_) were determined. Statistically significant differences between *A*_*z*_ values are reported as 95% confidence intervals. Statistical analyses were performed using SPSS for Windows (version 17.0.1; SPSS Inc., Chicago, IL) and *P* < 0.05 was considered statistically significant.

## 3. Results

**Figure 2:**
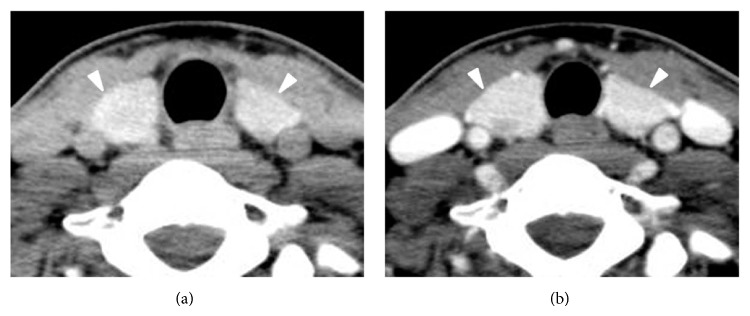
Normal thyroid designated “normal” CT diagnosis in a 28-year-old woman (papillary thyroid carcinoma in the left thyroid). The thyroid gland shows iso- and homogeneous attenuation in nonenhanced CT image (a) and anteroposterior diameter of 1-2 cm, smooth margin, and homogeneous enhancement in contrast-enhanced CT image (b).

**Figure 3:**
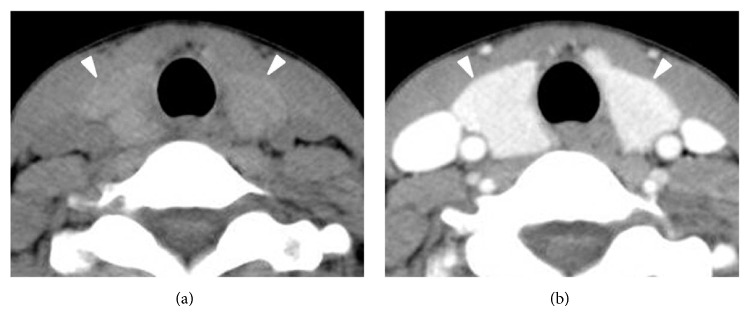
Non-Hashimoto lymphocytic thyroiditis designated “3 or more” CT classification in a 32-year-old woman (papillary thyroid carcinoma in the isthmus). The thyroid gland shows low and inhomogeneous attenuation in nonenhanced CT image (a) and mildly increased glandular size in contrast-enhanced CT image (b).

Of the 209 patients (170 women and 39 men; average age 47.1 ± 10.3 years; age range 22–75 years), the histopathology of the main surgical thyroid lesions included papillary thyroid carcinoma (*n* = 186), follicular thyroid carcinoma (*n* = 2), follicular adenoma (*n* = 7), and nodular hyperplasia (*n* = 14). The type of thyroid surgery included total thyroidectomy (*n* = 106), subtotal thyroidectomy (*n* = 16), and hemithyroidectomy (*n* = 87). Histopathological diagnoses of the 209 thyroid cases included normal thyroid (*n* = 157), Hashimoto thyroiditis (*n* = 17), non-Hashimoto lymphocytic thyroiditis (*n* = 34), and diffuse hyperplasia (*n* = 1) (Figures 2 and 3).

The frequencies of individual CT features of normal thyroid and incidental DTD in 209 patients are summarized in Table 1. The degree and pattern of parenchymal attenuation, glandular margin, and pattern of parenchymal enhancement showed a significant difference between normal thyroid and DTD, but there was no significant difference in glandular size.

**Figure 4:**
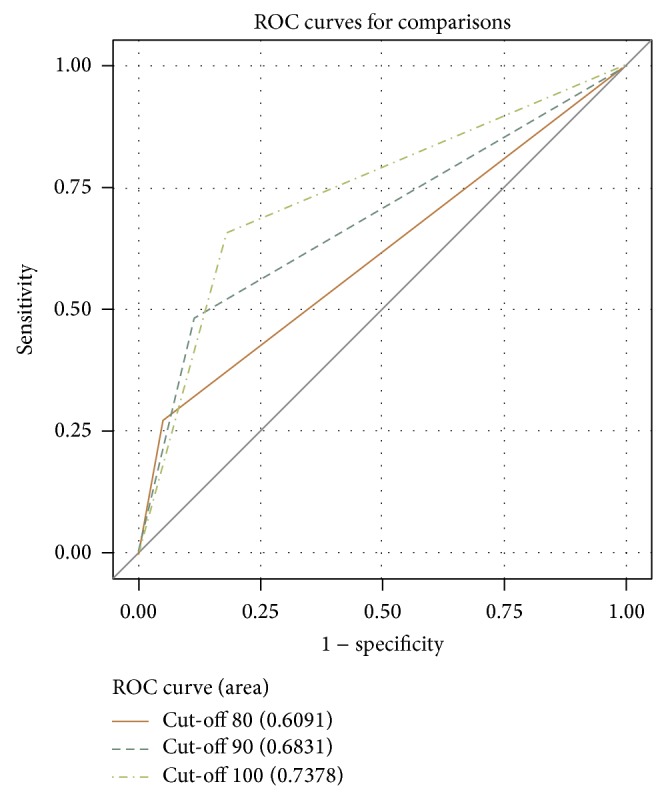
Receiver-operating characteristic (ROC) analysis for the cut-off Hounsfield unit (HU) values of the thyroid parenchyma in nonenhanced CT images. When 80, 90, or 100 HU is considered as a cut-off value, the *A*_*z*_ values are 0.6091 (unbroken line), 0.6831 (dashed line), and 0.7378 (dotted and dashed line), respectively. When 100 HU is determined as a cut-off value, the sensitivity, specificity, positive and negative predictive values, and accuracy of US diagnosis are 65.4%, 82.2%, 54.8%, 87.8%, and 78.0%, respectively.

The mean (±SD) HU values of the thyroid parenchyma on nonenhanced CT and contrast-enhanced CT were 114.3 (±21.2) and 202.5 (±29.3), respectively, in normal thyroid and 94.5 (±21.3) and 187.6 (±29.9), respectively, in DTD. ROC curve analysis showed that CT diagnosis of DTD using a cut-off value of <100 HU (i.e., decreased attenuation) in the thyroid parenchyma on nonenhanced CT was most effective compared with the other conditions (Table 2) (Figure 4) but revealed that there was no significant difference in cut-off value for the degree of parenchymal enhancement between normal thyroid and DTD. Diagnostic indices for individual CT features are shown in Table 3. The CT features suggestive of DTD included low attenuation, inhomogeneous attenuation, increased glandular size, lobulated margin, and inhomogeneous enhancement, while isoattenuation, homogeneous attenuation, anteroposterior diameter of 1-2 cm, smooth margin, and homogeneous enhancement were suggestive of normal thyroid.

**Figure 5:**
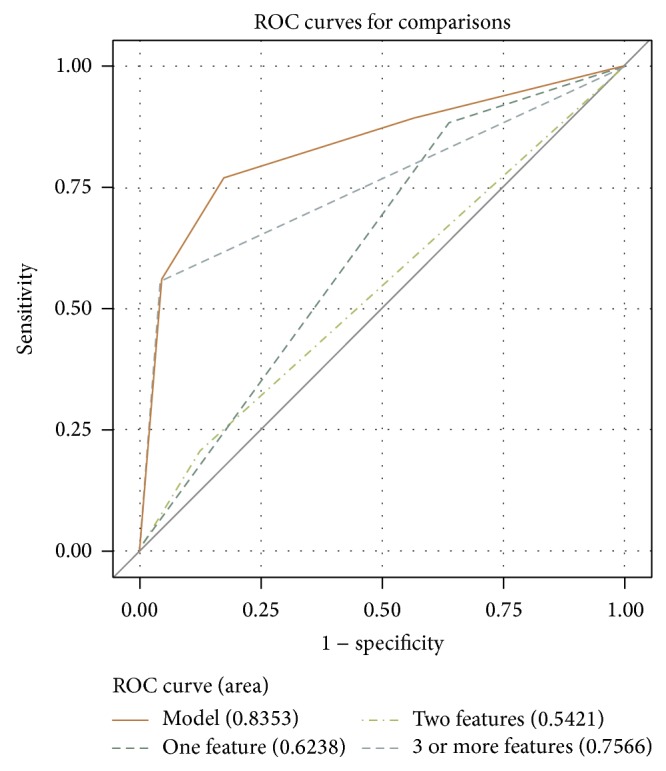
Receiver-operating characteristic (ROC) analysis for CT classification in the differentiation of diffuse thyroid disease (DTD) from normal thyroid. When the cases having “1 or more,” “2 or more,” and “3 or more” abnormal CT features are considered DTD, the *A*_*z*_ values are 0.6238 (short dashed line), 0.5421 (dotted and dashed line), and 0.7566 (long dashed line), respectively. When “3 or more” CT classification is selected, the sensitivity, specificity, positive and negative predictive values, and accuracy of CT diagnosis for DTD are 55.8%, 95.5%, 80.6%, 86.7%, and 85.6%, respectively.

Cases were categorized according to the number of abnormal CT features as follows: 1 or more (*n* = 98), 2 or more (*n* = 50), 3 or more (*n* = 31), 4 or more (*n* = 15), and 5 (*n* = 5). Comparison of the CT diagnoses and the histopathological diagnoses of the 209 thyroid cases revealed the following. The 111 “normal” CT cases included non-Hashimoto lymphocytic thyroiditis (*n* = 8) and normal thyroid (*n* = 103). The 98 cases with “1 or more” abnormal CT features included Hashimoto thyroiditis (*n* = 17), non-Hashimoto lymphocytic thyroiditis (*n* = 26), diffuse hyperplasia (*n* = 1), and normal thyroid (*n* = 54). The 50 cases with “2 or more” abnormal CT features included Hashimoto thyroiditis (*n* = 16), non-Hashimoto lymphocytic thyroiditis (*n* = 20), diffuse hyperplasia (*n* = 1), and normal thyroid (*n* = 13). The 31 cases with “3 or more” abnormal CT features included Hashimoto thyroiditis (*n* = 13), non-Hashimoto lymphocytic thyroiditis (*n* = 12), diffuse hyperplasia (*n* = 1), and normal thyroid (*n* = 5). The 15 cases with “4 or more” abnormal CT features included Hashimoto thyroiditis (*n* = 7), non-Hashimoto lymphocytic thyroiditis (*n* = 6), diffuse hyperplasia (*n* = 1), and normal thyroid (n = 1). In ROC curve analysis, the diagnostic accuracy of CT diagnosis for DTD was the highest when the “3 or more” CT classification was applied (Figure 5). When the “3 or more” CT classification was selected, the sensitivity, specificity, positive and negative predictive values, and accuracy of CT diagnosis for incidental DTD were 55.8%, 95.5%, 80.6%, 86.7%, and 85.6%, respectively (Table 4).

## 4. Discussion

Symptomatic DTD is easily diagnosed by clinicoserologic examination, but reliable diagnostic techniques for detection of asymptomatic or subclinical DTD have not been established [4–7]. DTD is known to be associated with a high incidence of thyroid malignancy; thus, regular US follow-up in patients with DTD may be required [11, 12]. The role of imaging-based diagnosis for DTD is controversial, but our previous study using real-time thyroid US for diagnosis of incidental DTD revealed the sensitivity, specificity, positive and negative predictive values, and accuracy as 87.7%, 92.1%, 70.4%, 97.2%, and 91.3%, respectively [4]. In the present study, when a CT classification of “3 or more” abnormal CT features was applied, the sensitivity, specificity, positive and negative predictive values, and accuracy of CT diagnosis for incidental DTD were 55.8%, 95.5%, 80.6%, 86.7%, and 85.6%, respectively. Compared with real-time US, CT diagnosis of incidental DTD showed similar diagnostic values, except for sensitivity.

Based on the literature, sonographic features suggestive of DTD include decreased or increased parenchymal echogenicity, coarse echotexture, decreased or increased vascularity, decreased or increased anteroposterior diameter of the thyroid gland, the presence of marginal nodularity, the presence of scattered microcalcifications, “thyroid inferno,” and “micronodulation” [13–15]. In our study, individual CT features were selected according to previously established sonographic features, but no CT feature showed both high sensitivity and specificity. The CT features that exhibited a high specificity and low sensitivity included low attenuation, inhomogeneous attenuation, increased glandular size, lobulated margin, and inhomogeneous enhancement.

Accurate measurement of CT HU values for the thyroid gland can be achieved by measurement of the entire thyroid gland, but this is impractical. Instead, we used ROIs with a round shape in a PACS and measured CT HU values in both thyroid lobes on nonenhanced and contrast-enhanced CT images. In nonenhanced CT, the cut-off HU value between normal thyroid and DTD was 100 HU. Namely, a CT HU value of <100 in the thyroid parenchyma on nonenhanced CT was suggestive of DTD. However, no cut-off value was established between normal thyroid and DTD in the degree of parenchymal enhancement. The reason for this is not clear, but enhancement variations according to timing of CT after injection of contrast medium and the interference of iodine contents within the thyroid gland might have been factors in this finding.

A CT classification of “3 or more” abnormal CT features was more valuable in CT diagnosis for DTD than “1 or more” and “2 or more.” However, only 30 (55.6%) cases with DTD had 3 or more abnormal CT features. Namely, “3 or more” CT classification showed a low sensitivity despite a high specificity and high positive predictive value. Therefore, further study regarding CT criteria may be required.

There are several limitations to this study. First, all the study patients underwent thyroid surgery. This condition was necessary for using the histopathological results of the thyroid as reference standard, but sampling bias might be possible. Second, only a single radiologist performed image analysis of neck CT. Third, clinicoserologic data were not included in this study. Therefore, Graves' disease was not distinguished from DTD. Fourth, histopathological thyroid diagnoses were determined by a single pathologist. Finally, we did not evaluate the difference between the 2 CT modalities used in this study.

In conclusion, the study results showed that CT features suggestive of DTD included low attenuation, inhomogeneous attenuation, increased glandular size, lobulated margin, and inhomogeneous enhancement and that CT diagnosis of DTD using the “3 or more” CT classification was superior. Therefore, neck CT may be helpful for the detection of incidental DTD. In addition, most of useful CT features for differentiating DTD from normal thyroid were detected in nonenhanced CT images.

## Figures and Tables

**Table 1:** Frequency analysis of CT features of normal thyroid and diffuse thyroid disease in 209 patients.

CT features	Normal thyroid	Diffuse thyroid disease	*P* value
Degree of attenuation^*^			
Iso-	130 (82.8)	18 (34.6)	<0.0001
Low	27 (17.2)	34 (65.6)	
High	0 (0)	0 (0)	
Pattern of attenuation			
Homogeneous	142 (90.4)	17 (32.7)	<0.0001
Inhomogeneous	14 (8.9)	35 (67.3)	
Heterogeneous	1 (0.6)	0 (0)	
Size of thyroid gland			
Normal	134 (85.4)	38 (73.1)	0.0749
Increased	22 (14.0)	14 (26.9)	
Decreased	1 (0.6)	0 (0)	
Margin of thyroid gland			
Smooth	153 (97.5)	40 (76.9)	<0.0001
Lobulated	4 (2.5)	12 (23.1)	
Pattern of enhancement			
Homogeneous	152 (96.8)	22 (42.3)	<0.0001
Inhomogeneous	5 (3.2)	30 (57.7)	
Heterogeneous	0 (0)	0 (0)	

Data are number of cases, with percentage in parentheses. ^*^According to the measured HU value in the thyroid parenchyma on nonenhanced CT, the degree of parenchymal attenuation was classified as iso- (from 100 HU to 180 HU), low (<100 HU), and high (>180 HU).

**Table 2:** Diagnostic indices of a cut-off value of parenchymal Hounsfield unit in nonenhanced CT for detecting diffuse thyroid disease in 209 patients.

Cut-off HU value	Sensitivity (%)	Specificity (%)	PPV (%)	NPV (%)	Accuracy (%)	ROC (AUC)	95% confidence interval
80 or less	14/52 (26.9)	149/157 (94.9)	14/22 (63.6)	149/187 (79.7)	163/209 (78.0)	0.6091	0.2692–0.9490
90 or less	25/52 (48.1)	138/157 (88.5)	25/43 (58.1)	138/165 (83.6)	163/209 (78.0)	0.6831	0.4808–0.8846
100 or less	34/52 (65.4)	129/157 (82.2)	34/62 (54.8)	129/147 (87.8)	163/209 (78.0)	0.7378	0.6538–0.8217

HU: Hounsfield unit; PPV: positive predictive value; NPV: negative predictive value; ROC: receiver-operating characteristic analysis; AUC: area under curve.

**Table 3:** Diagnostic index of individual CT features for detecting diffuse thyroid disease in 209 patients.

CT features	Sensitivity (%)	Specificity (%)	PPV (%)	NPV (%)	Accuracy (%)
Degree of attenuation					
Iso-	18/52 (34.6)	27/157 (17.2)	18/148 (12.2)	27/61 (44.3)	45/209 (21.5)
Low	34/52 (65.4)	130/157 (82.8)	34/61 (55.7)	130/148 (87.8)	164/209 (78.5)
Pattern of attenuation					
Homogeneous	17/52 (32.7)	15/157 (9.6)	17/159 (10.7)	15/50 (30)	32/209 (15.3)
Inhomogeneous	35/52 (67.3)	143/157 (91.1)	35/49 (71.4)	143/160 (89.4)	178/209 (85.2)
Heterogeneous	0/52 (0)	156/157 (99.4)	0/1 (0)	156/208 (75)	156/209 (74.6)
Size of thyroid gland					
Normal	38/52 (73.1)	23/157 (14.7)	38/172 (22.1)	23/37 (62.2)	61/209 (29.2)
Increased	14/52 (26.9)	135/157 (86.0)	14/36 (38.9)	135/173 (78.0)	149/209 (71.3)
Decreased	0/52 (0)	156/157 (99.4)	0/1 (0)	156/208 (75)	156/209 (74.6)
Margin of thyroid gland					
smooth	40/52 (76.9)	4/157 (2.5)	40/193 (20.7)	4/16 (25)	44/209 (21.1)
Lobulated	12/52 (23.1)	153/157 (97.5)	12/16 (75)	153/193 (79.3)	165/209 (78.9)
Pattern of enhancement					
Homogeneous	22/52 (42.3)	5/157 (3.2)	22/174 (12.6)	5/35 (14.3)	27/209 (12.9)
Inhomogeneous	30/52 (57.7)	152/157 (96.8)	30/35 (85.7)	152/174 (87.4)	182/209 (87.1)

Data in parentheses are percentage of each item. PPV: positive predictive value; NPV: negative predictive value.

**Table 4:** Diagnostic indices of CT detection for diffuse thyroid disease in 209 patients according to the number of individual CT features.

CT classification	Sensitivity (%)	Specificity (%)	PPV (%)	NPV (%)	Accuracy (%)	ROC (AUC)	95% confidence interval
1 or more	46/52 (88.5)	73/157 (46.5)	46/130 (35.4)	73/79 (92.4)	119/209 (56.9)	0.6238	0.5660–0.6817
2 or more	40/52 (76.9)	130/157 (82.8)	40/67 (59.7)	130/142 (91.5)	170/209 (81.3)	0.5421	0.4802–0.6039
3 or more	29/52 (55.8)	150/157 (95.5)	29/36 (80.6)	150/173 (86.7)	179/209 (85.6)	0.7566	0.6865–0.8266

PPV: positive predictive value; NPV: negative predictive value; ROC: receiver-operating characteristic analysis; AUC: area under curve.
